# Adsorption and safe immobilization of Sr ions in modified zeolite matrices

**DOI:** 10.1038/s41598-023-46381-9

**Published:** 2023-11-04

**Authors:** Mahya Fayezi, Zahra Shiri-Yekta, Hamid Sepehrian, Mehran Heydari, Mohammad Rahghoshay, Samaneh Zolghadri

**Affiliations:** 1 Department of Nuclear Engineering, Science and Research Branch, Islamic Azad University, P. O. Box: 14515-775, Tehran, Iran; 2 Nuclear Fuel Cycle Research School, Nuclear Science and Technology Research Institute, P.O. Box: 11365-8486, Tehran, Iran; 3 Radiation Application Research School, Nuclear Science and Technology Research Institute, P.O. Box: 14155-1339, Tehran, Iran

**Keywords:** Chemistry, Nuclear chemistry

## Abstract

In the present study, an Iranian natural zeolite (Sabzevar region) was evaluated as a natural adsorbent for the elimination and immobilization of strontium ions from an aqueous solution. For improving the adsorption efficiency of strontium ion, the zeolite surface was modified by the Schiff base ligand of bis (2-hydroxybenzaldehyde)1,2-diaminoethane (H_2_L). The natural zeolite and zeolite/H_2_L were characterized using Fourier transform infrared spectroscopy (FT-IR), X-ray diffraction (XRD), X-ray fluorescence (XRF), BET and scanning electron microscope (SEM). Analysis of the natural zeolite showed that the zeolite is from the type of clinoptilolite and has a crystalline structure with the specific surface area 29.74 m^2^/g. The results showed that strontium adsorption onto modified zeolite increases compared to unmodified zeolite from 64.5% to 97.2% (at pH = 6). The effective parameters pH, adsorbent dosage, initial concentration of strontium ions, contact time, temperature, and interfering ions, were studied and optimized. The maximum adsorption efficiency was confirmed by modified zeolite and found to be 97.5% after 60 min of equilibrium time at pH 6, 0.05g as adsorbent dosage, and at 25 °C. Adsorption of strontium was confirmed by Langmuir model with maximum adsorption capacity of 10.31 mg/g. Kinetic studies showed that the adsorption of strontium ions on the adsorbent follows pseudo-second-order (PSO) model. Also, the thermodynamics of the adsorption process indicated that the adsorption of strontium on zeolite/H_2_L is an endothermic and spontaneous process, and the adsorption mechanism is a combination of physical and chemical adsorption. Finally, to manage the secondary waste generated from the adsorption process, strontium ions were immobilized in a zeolite structure. The results showed that the stabilization is well done with the thermal preparation process. After thermal treatment at 25–900 °C, modified zeolite satisfactorily retains strontium during back-exchange tests with NaCl solution. According to the results, the amount of strontium released from the adsorbent phase decreases from 52.6 to 1.6% with increasing heat treatment temperature.

## Introduction

Many nuclear industry activities produce radioactive waste in the environment^1^. Usually, the complete inhibition of waste production in various sectors, such as the nuclear industry, is not possible despite the use of the best techniques for reducing produced waste, and significant amounts of waste generated during the operation and exploitation. It should be noted that the operation of nuclear waste management is the same general way used everywhere, but the basic principles governing the operation of waste management to preserve humans and the environment from any harmful effects of radioisotopes and ionizing radiations are identical^2^.

Strontium is an alkaline element and like other members of the family, especially calcium, barium, and radium. Strontium has an atomic radius similar to calcium and easily replaces calcium in minerals. ^90^Sr is one of the essential components of several nuclear wastes, which is a fusion product of ^235^U^3^. This isotope (^90^Sr) is one of the most dangerous nuclear fission products for humans^4^. Radioactive strontium can compete with calcium in the biosphere, known as a bone seeker, and may also be transferred to the human body via the food chain due to its long retention time. ^90^Sr is adsorbed by the digestive system, where it congregates in the bone marrow tissue and damages the blood-producing cells. It may also cause leukemia or skeletal cancer^4^. So, the separation and removal of strontium ions from aqueous solutions is very important.

Ion exchangers are widely used to treat industrial wastewater and valuable metals are recovered at a lower cost than conventional chemical treatment with significant savings in treatment plant space. Various sorbents/ion exchangers are described in the literature for the adsorption of strontium, cesium, and uranium. Synthetic and natural adsorbents and inorganic ion exchangers, such as sepiolite^5^, kaolin^6^, titanosilicates and titanates^7–9^, metal oxides and their mixtures^10–13^, and acidic salts of polyvalent metals, salts of heteropolyacids^14,15^, compared to the known organic resins, have advantages in terms of higher chemical and thermal stability. In addition, they have specific selectivity for some ions.

Zeolites are another valuable inorganic crystalline material that has many industrial applications, especially in the treatment of radioactive liquid wastes because of their structural characteristics such as high mechanical and chemical resistance, porosity, and the presence of alkaline and earth alkaline metal cations. They offer good cation exchange, and catalytic properties and are desirable for analytical purposes^4,16–20^.

Furthermore, if the cation exchange reaction facilitates the treatment of nuclear power plant wastewater, it is evident that the waste problem is subsequently moved to contaminated zeolites, which should be reserved in an appropriate disposal tank as radioactive waste. The greatest conventional processes of stabilization of these wastes include freezing with inorganic reactants, immobilization in a cement matrix, vitrification, and caramelization. Stabilizing the generated waste in an appropriate matrix is typically an effective process to safely and irreversibly entrap the cation. This method has been demonstrated to be proper to eliminate and securely dispose of strontium and cesium ions either through natural or synthetic zeolites^21–26^. Heat treatment at high temperatures causes the zeolite framework to break down and form an amorphous phase, "encapsulating" the unwanted species.

One of the most important characteristics related to nuclear energy is radioactive waste management and disposal. Immobilization of radioactive elements should be planned to ensure that there is no significant release to the environment. So, the present research focused on using clinoptilolite from Iranian zeolites in the Sabzevar region. First, the natural zeolite was prepared and characterized. Then natural zeolite was modified by Schiff base ligand bis (2-hydroxybenzaldehyde)1,2-diaminoethane (H_2_L) to improve the removal and separation of strontium ions. Adsorption of this ion on the surface of zeolite showed the pH dependency. It is noteworthy that with the modification of zeolite by the H_2_L ligand, a significant increase in the adsorption percentage of strontium ions from aqueous phases was observed (at pH = 6 from 64.5% to 97.2%). Other parameters affecting to adsorption properties of the modified zeolite were investigated and the adsorption of strontium ions on the adsorbent was evaluated in terms of equilibrium, kinetic, and thermodynamic studies. Then, the strontium ion loaded onto the modified adsorbent was immobilized by the thermal treatment method. The leaching test results showed that the consolidation rate is improved with increasing temperature.

## Experimental

### Materials and instruments

The zeolite Sabzevar mines were supplied by a company (afrand tooska) in Tehran, Iran. Schiff base ligand bis (2-hydroxybenzaldehyde)1,2-diaminoethane (H_2_L) was synthesized by using 2-hydroxybenzaldehyde and ethylendiamine (Merck) in methanol solvent for modification of zeolite. To prepare stock solutions of strontium ion (1000 mg/L), an appropriate amount of strontium nitrate salt was dissolved in distilled water. All the other reagents and chemicals used were of analytical grade and were attained from Merck or Aldrich.

The pH is adjusted with a Metrohm (model 780) by adding nitric acid or sodium hydroxide (0.1 M). A water bath incubator shaker Infors AG (model Aquatron) was applied for mixing adsorbent and aqueous solutions. Abstraction of the adsorbent from the solution was conducted by a Sigma centrifuge (model 3-30K). A Varian Liberty 150 XL inductively coupled plasma (ICP) was employed for the analysis of metal ions. The FT-IR spectra natural zeolite and modified zeolite before and after adsorption of strontium ion was recorded using a Brucker Vector 22 spectrophotometer using KBr disks. Adsorbance was measured as a function of the wavenumber (cm^−1^) between 400 and 4000 cm^−1^. X-ray powder diffraction (XRD) was accomplished with a 3710 PW Philips in the range of 2θ between 5 and 70°. The surface analysis of natural zeolite before and after modification with H_2_L ligand were determined using BET (Quantachrome Instruments, version 2.2). Surface morphology natural zeolite and modified zeolite before and after adsorption of strontium ion was visualized by the scanning electron microscopy (SEM, Philips XL-30). The chemical composition of zeolite was also defined by the X-Ray fluorescence (XRF, Oxford ED2000), and a furnace (Exiton, model Atash-1200) was employed for immobilizing the component of loaded strontium onto modified zeolite in different temperatures.

### Synthesis and characterization of ligand

**Figure 1:**
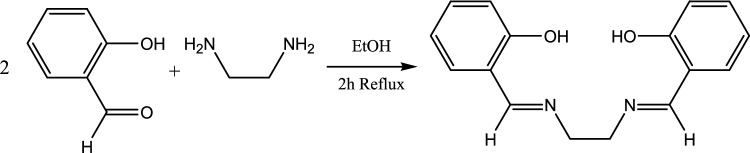
Synthesis of the bis (2-hydroxybenzaldehyde)1,2-diaminoethane Ligand (H_2_L).

The H_2_L ligand was prepared similarly to the previous methods^27^. The schematic of H_2_L ligand synthesis is shown in Fig. 1. 1.23 g 2-hydroxybenzaldehyde (0.01 mol) in 30 mL of ethanol solution was refluxed with 0.39 g of ethylenediamine (0.005 mol) for 2 h. The deposited compound was separated by a filter, and washed with ethanol and water many times: yield, 1.27 g (78.0%). IR: ν_C=C_ = 1578 cm^−1^, ν_C=N_ = 1636 cm^−1^, ν_C-H_ = 2902 cm^−1^, ν_O-H_ = 3450 cm^−1^^27^.

### Zeolite modifying

The modification of the natural zeolite was conducted based on the following order: (a) 5 g of the zeolite was dehydrated at 100 °C; (b) then, this amount of adsorbent was refluxed with 1 g ligand in dichloromethane solvent for 24 h; (c) for exiting the excess ligands that were not entered in the structure of the zeolite, the adsorbent produced in the previous step was soxhlet until the solvent was colorless; (d) the adsorbent obtained was filtered and washed many times with distilled water to eliminate the solvent; (e) then, the modified zeolite was dried at 70 °C.

### Adsorption procedure

Adsorption runs were performed in polyethylene containers in a shaker water bath using 0.05 g of the adsorbents in 20 mL of sample including metal ion and stirred for 60 min. Filter-separating of solid phase from liquid was followed by centrifuging at 5000 rpm for 10 min. The concentration of cation remaining in the aqueous phase was defined by ICP, and the amount of adsorbed ions was estimated from the change between the initial and final concentrations of cation.

The adsorption and desorption percentage of strontium ion from aqueous solution was calculated as follows^28^:

1 $$ {\text{Adsorption\% }} = \frac{{\left( {{\text{C}}_{i} - {\text{C}}_{{\text{f}}} } \right)}}{{{\text{C}}_{{\text{i}}} }} \times {100,} $$

2 $$ {\text{Desorption\% }} = \frac{{{\text{m}}_{{\text{d}}} }}{{{\text{m}}_{{\text{a}}} }} \times {100,} $$

where C_i_ and C_f_ are the initial and final strontium concentration (mg/L), respectively. ^“^m_d_^”^ and ^“^m_a_^”^ is the desorption and adsorption of ions from the adsorbent surface (mg), respectively.

### Immobilization and leaching tests

The safety test of trapping strontium in modified zeolite was performed in two stages:

- *Immobilizing test*: To test the safety of strontium trapping, first 1 g of the modified adsorbent disperses in 50 mL of 600 mg/L Sr(NO_3_)_2_ solution for 24 h. Then adsorber was separated from the liquid phase, and the amount of adsorbed ions by ICP was estimated. 0.2 g pellets of the Sr-zeolites were made using a hydraulic press with a loading pressure of 400 g/cm^2^ and heated at temperatures of 25, 60, 300, 600, and 900 °C for 4 h.
- *Leaching test*: Thermally treated tablet samples, after being powdered, were stirred for 24 h with 50 ml of a 1 M NaCl solution to study the quantity of back exchangeable strontium. Then, the liquid was removed from the powder by centrifugation and analyzed by ICP.

## Results and discussion

### Characterization

**Figure 2:**
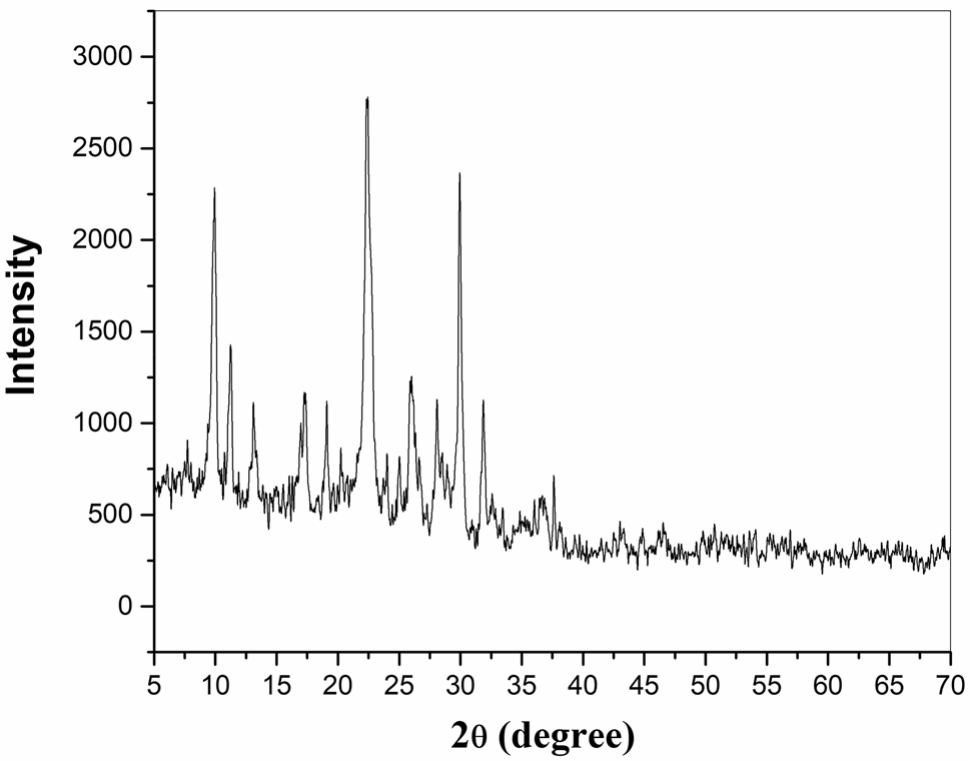
XRD patterns of the natural zeolite.

X-ray diffraction spectra of the natural zeolite was shown in Fig. 2. XRD analysis showed that the zeolite is from the type of clinoptilolite (Na–K) and has a crystalline structure. The crystal phase structure and crystal size of samples were examined by X-ray powder diffractometer using Cu K_α_ radiation (λ = 1.5405 Å). The crystallite size equal to 29.49 nm was defined from the characteristic peak at 2θ = 22.36° for natural zeolites using the Scherrer formula^29^:

3 $$ {\text{nm}} = {\text{K}}\lambda /{\text{Wcos}}\theta , $$

where K, the shape factor = 0.9, λ = wavelength of the X-ray used, and W = width of peak at half-height. Also, XRF analysis of the natural zeolite and the details of their percentage chemical composition were mentioned in Table 1. The specific surface area (S_BET_) and pore volume of natural zeolite and modified zeolite are reported in Table 2. As shown in the table, the specific surface area of natural zeolite is 29.74 m^2^/g that after modification of natural zeolite using H_2_L ligand the specific surface area of natural zeolite was decreased by 12.6%. The modification of natural zeolite may have resulted in pore blockage, leading to a decrease in specific surface area, but this modification also introduced additional adsorption sites, thereby enhancing the adsorption capacity of the modified zeolite^19^.

**Table 1:** XRF chemical analysis results of natural zeolite.

Oxides	Content of Iranian natural zeolite (%)
SiO_2_	68.47
Al_2_O_3_	9.19
CaO	0.61
K_2_O	1.71
Fe_2_O_3_	0.79
Na_2_O	4.07
TiO_2_ and MnO	< 1

**Table 2:** The BET analysis of natural zeolite and modified zeolite.

Adsorbent	BET surface area (m^2^/g)	Total pore volume (cm^3^/g)
Natural zeolite	29.74	9.76
Modified zeolite	25.98	8.53

**Figure 3:**
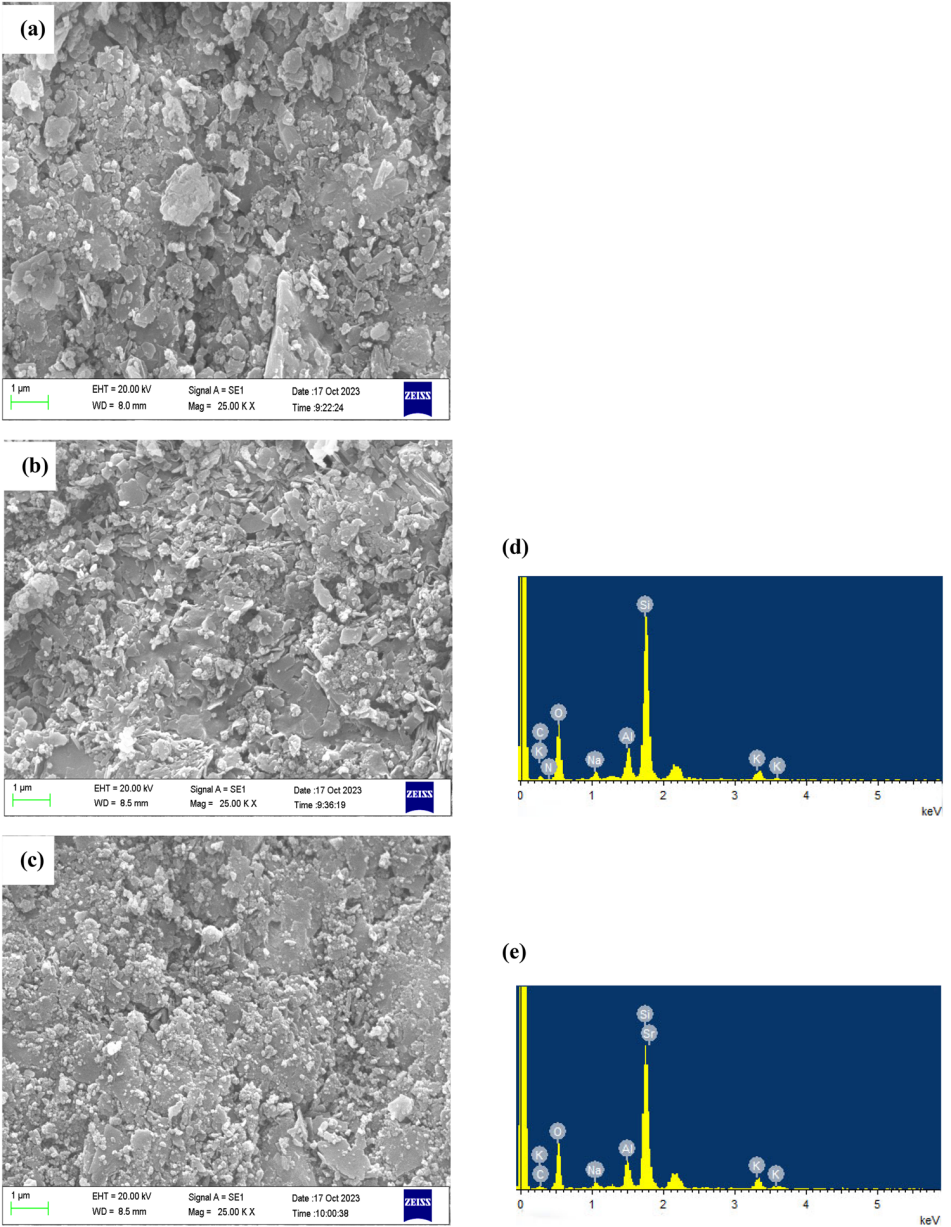
SEM of (**a**) natural zeolite and modified zeolite (**b**) before and (**c**) after the adsorption of strontium. The EDX spectrum of modified zeolite (**d**) before and (**e**) after the adsorption of strontium.

Figure 3a,b shows the SEM images of the natural and modified zeolites. The micrographs indicated that there were no significant changes in the surface morphology of natural zeolites after the modification process. Figure 3c shows the surface morphology of modified zeolite after strontium adsorption. Figure 3d,e present the EDX spectrum of modified zeolite before and after strontium adsorption. The results of the EDX analysis show the presence of the C and N elements in the modified zeolite with H_2_L ligand structure (Fig. 3d). These results provide strong evidence of the successful modification of the natural zeolite surface. The adsorption of strontium on modified zeolite is confirmed by EDX analysis (as presented in Fig. 3e). It shows the presence of strontium at approximately 1.8 keV, confirming the adsorption of strontium onto the surface of modified zeolite.

**Figure 4:**
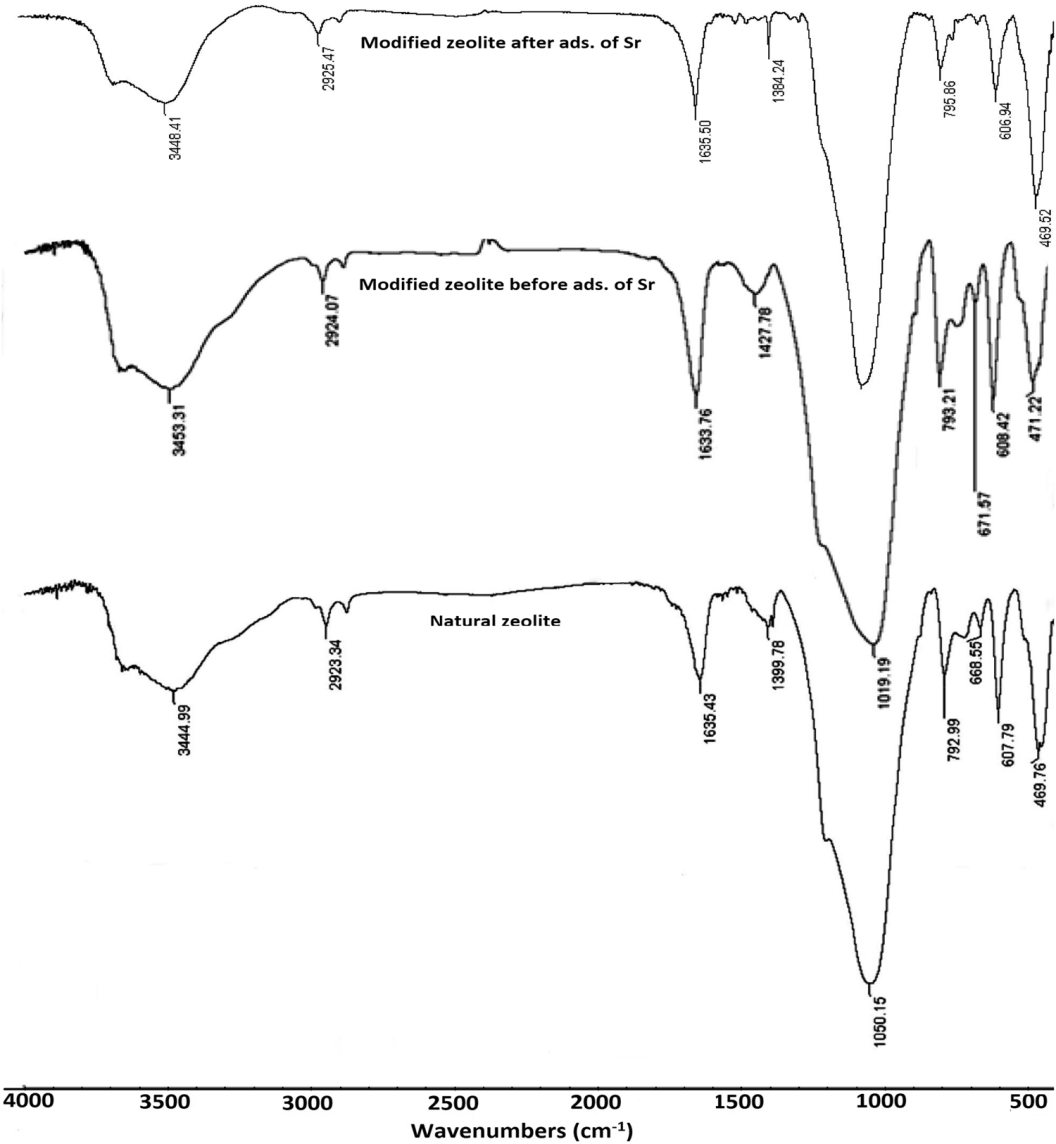
The FTIR spectrum of natural zeolite and modified zeolite (before and after the adsorption of strantium).

Figure 4 shows the FT-IR spectra of the natural zeolite and zeolite/H_2_L before and after adsorption of strontium ion. In the 400–600 cm^-1^ region, the band observed is related to the double ring in the natural zeolite structure. The vibration frequencies at 1050, and 793 cm^-1^ were allocated to the asymmetric and symmetric stretching related to the external bonds, respectively^30,31^. The peaks at 3100–3700 and 1400–1700 cm^-1^ are related to interstitial water and hydroxyl group, and the deformation vibration of the free water molecules, correspondingly^32^. In the FT-IR spectrum of zeolite/H_2_L, the intensity of the peaks in the regions of 1633 and 3100–3700 cm^−1^ has increased, which corresponds to the vibration frequencies of C = N and O–H groups in the ligand structure. After the adsorption of strontium ions on modified zeolite, the peaks at the 1200–1500 cm^-1^ region almost disappeared. The majority of bands were shifted, confirming the adsorption of strontium onto modified zeolite.

### Adsorption runs

#### Effect of pH

**Figure 5:**
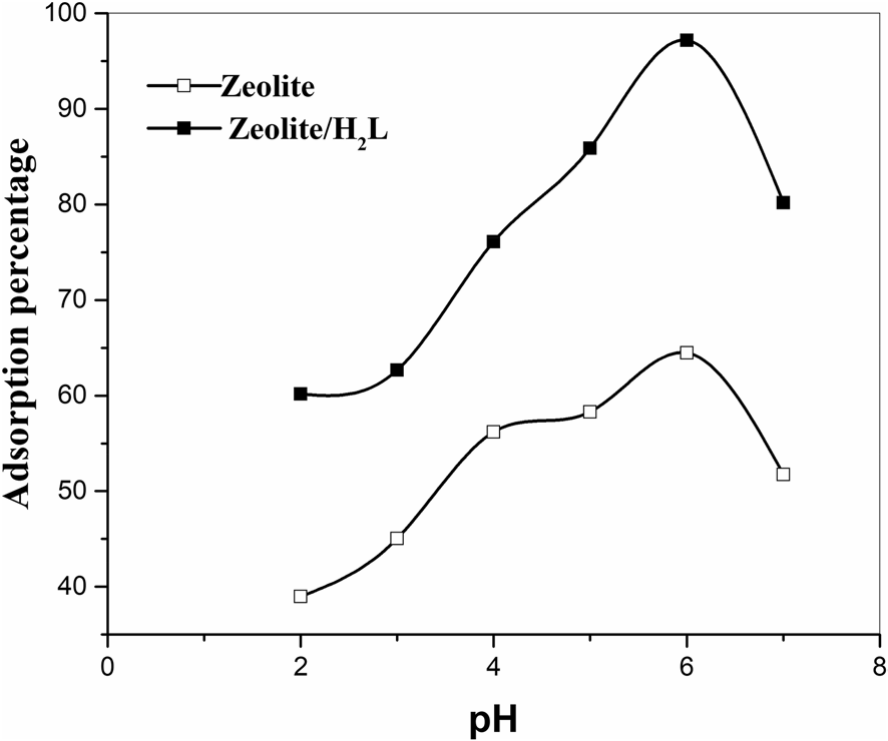
Impact of pH on the adsorption of strontium ions by zeolite and zeolite/H_2_L in experimental conditions: shaker speed: 150 rpm, temperature: 25°C, amount of adsorbent: 0.05 g, contact time: 60 min, initial metal concentration: 20 mg/L, solution volume: 20 ml.

The pH is one of the decisive factors in the amount of adsorption capability. Comparison of the adsorption process of unmodified and modified zeolite showed that in addition to increasing the adsorption of strontium ions with increasing the pH, the presence of Schiff base ligand in zeolite structure was influential on the adsorption efficiency. So that with increasing pH, the adsorption amount increases significantly from 64.5% to 97.2% at pH = 6 (Fig. 5). The results show that the adsorption in an alkaline medium is somewhat higher than in an acidic medium, which is a general phenomenon for most inorganic ion exchangers^33^. This behavior may arise from the competition between strontium and hydrogen ions for adsorption on the adsorbents. From this figure, it was seen that the adsorption percentage of strontium continuously improves with intensifying pH, and the highest adsorption is attained at pH = 6. Therefore, pH = 6 was selected as the optimal pH, and other factors were investigated using the modified zeolite.

#### Effect of adsorbent amount

**Figure 6:**
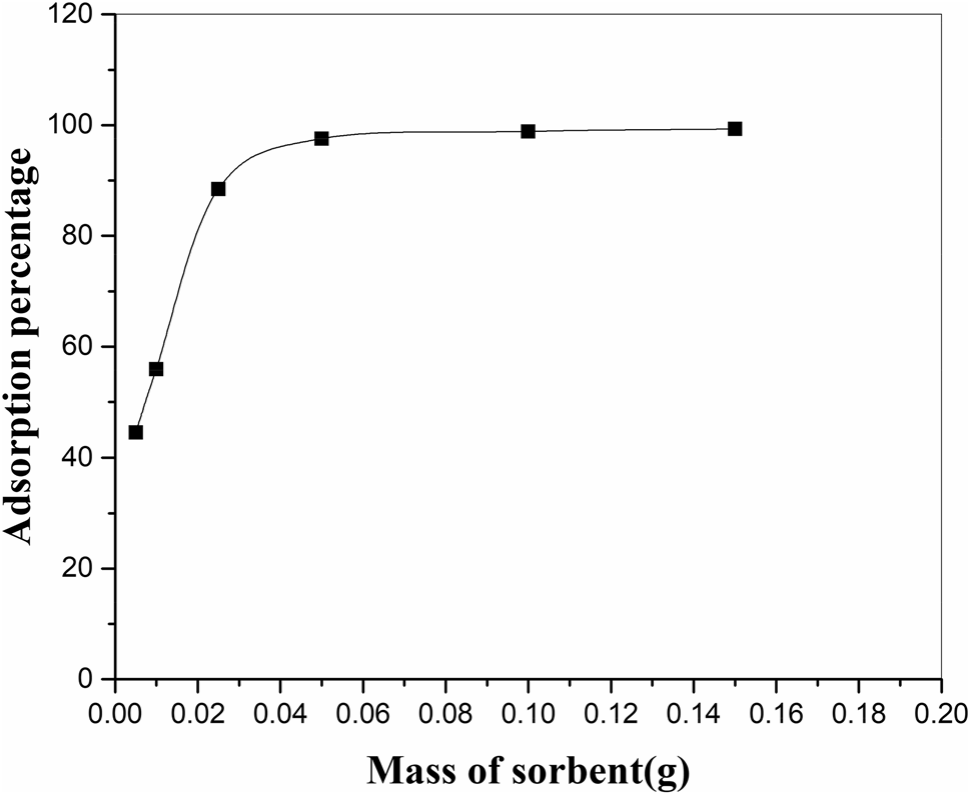
Impact of adsorbent amount on strontium adsorption by adsorbent zeolite/H_2_L in experimental conditions: pH = 6, shaker speed: 150 rpm, temperature: 25 °C, contact time: 60 min, initial metal concentration: 20 mg/L, solution volume: 20 ml.

To examine this parameter, 0.005–0.15g of the modified adsorbent was used for the sorption of 20 mg/L of strontium ions from a 20 mL solution. Figure 6 shows that with the increasing amount of adsorbent, the adsorption efficiency of strontium ions increases because of the increase of adsorption parts^34^. An additional increment in the amount of adsorbent beyond 0.05 g did not change the outcomes, representing surface saturation and equilibrium between the adsorbent and ions. A quantitative uptake (> 97%) of strontium ions was achieved using 0.05 g of the modified zeolite.

#### Influence of shaking time

**Figure 7:**
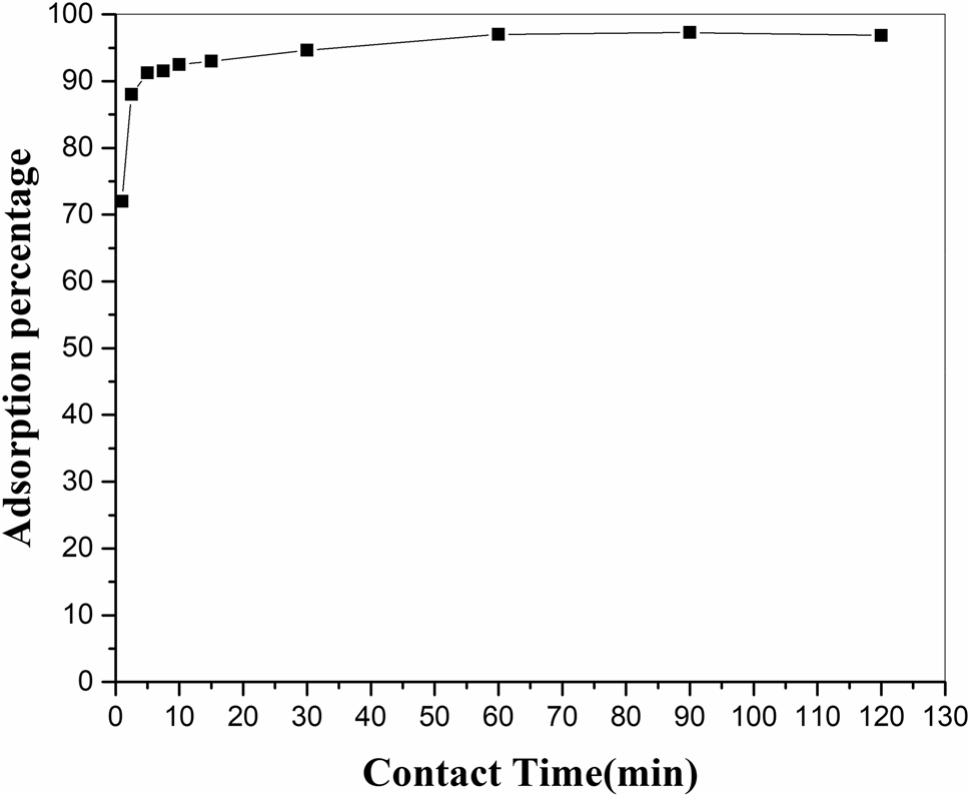
Effect of contact time on the adsorption of strontium ion by adsorbent zeolite/H_2_L in experimental conditions: pH = 6, shaker speed: 150 rpm, temperature: 25°C, amount of adsorbent: 0.05 g, initial metal concentration: 20 mg/L, solution volume: 20 ml.

To determine the impact of contact time between adsorbent and aqueous solution on strontium adsorption, differences in uptake percentage of strontium against time were sketched. The adsorption of strontium ions from solution with pH = 6 onto modified zeolite (0.05 g) was investigated with various shaking times in 5 to 120 min (Fig. 7). It was seen that the adsorption of strontium ions from aqueous solution via the modified adsorbent was rapid and enhanced continuously with increasing time until equilibrium between the two phases was achieved after 60 min. Hence, this obtained equilibrium time was used for subsequent adsorption tests.

#### Impact of initial concentration

**Figure 8:**
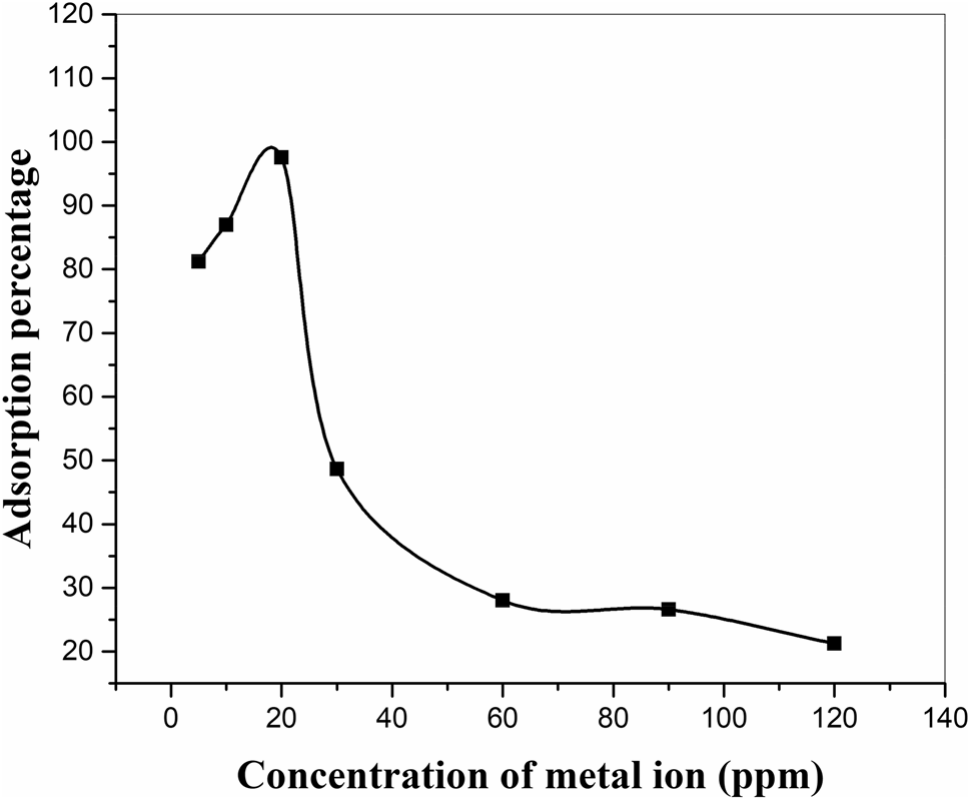
Effect of initial concentration of the strontium ion by adsorbent zeolite/H_2_L in experimental conditions: pH = 6, shaker speed: 150 rpm, temperature: 25 °C, amount of adsorbent: 0.05 g, contact time: 60 min, solution volume: 20 ml.

To investigate the maximum quantity of metal ion adsorbed by a certain extent of sorbent, this variable was investigated with 0.05 g of sorbent for initial concentrations ranging from 10 to 120 mg/L at pH = 6 (Fig. 8). The results show that by increasing the initial concentration up to 20 mg/L, the adsorption percentage increased. However, after the concentration of 20 mg/L, the relative sites available for adsorption on the surface of the adsorbent were low, and consequently, the abstraction percentage of metal ions decreased^35^.

#### Temperature dependency

The adsorption mechanism can be influenced by temperature. To evaluate the impact of this factor, the removal of strontium ions onto modified adsorbent was studied in the range of 10–45 °C, while other factors were maintained constant (20 mg/L of strontium in pH = 6). Table 3 shows the adsorption percentage of strontium ions on the adsorbent at different temperatures. This indicates that the uptake of strontium ions increases with increasing temperature from 10 to 25 °C, and the adsorption of strontium ion on adsorbent is endothermic process. Therefore, the high temperature is beneficial for strontium adsorption. The activity of strontium ions in solution increased with increasing temperature, which facilitated the coordination of strontium ions to functional groups of adsorbent surfaces^36^. The next experiments were performed at 25 °C. Above this temperature, adsorption decreases. These data can be illuminated by studying physical and chemical adsorption mechanisms^28^.

**Table 3:** The impact of temperature on the uptake values of strontium ion for zeolite/H_2_L.

Temperature (°C)	Uptake%
10	71.2
15	73.9
20	75.3
25	97.5
35	76.8
45	62.5

#### The effect of interfering ions

Because of the existence of other interfering ions in the nuclear waste, competitive adsorption of Co(II), Fe(III), Ni(II), Cs(I), and Na(I) ions with Sr(II) ion was performed by 0.05 g of adsorbent (Table 4). For this study, 20 ml of solution involving an initial concentration of 0.001 molar from Sr(II), Co(II), and Cs(I) ions, 0.002 molar of Fe(III) and Ni(II), and 0.005 molar of Na(I) ion in the presence of 0.05 g of adsorbent zeolite/H_2_L at pH = 6 was studied. The results showed that strontium ion has a higher adsorption than other ions, and the selectivity remains unchanged.

**Table 4:** The impact of interfering ions in adsorption of strontium ion onto zeolite/H_2_L.

Metal ions	Uptake%
Sr(II)	67.0
Fe(III)	16.8
Cs(I)	4.7
Co(II)	3.0
Ni(II)	2.6
Na(I)	2.0

### Adsorption kinetics

**Figure 9:**
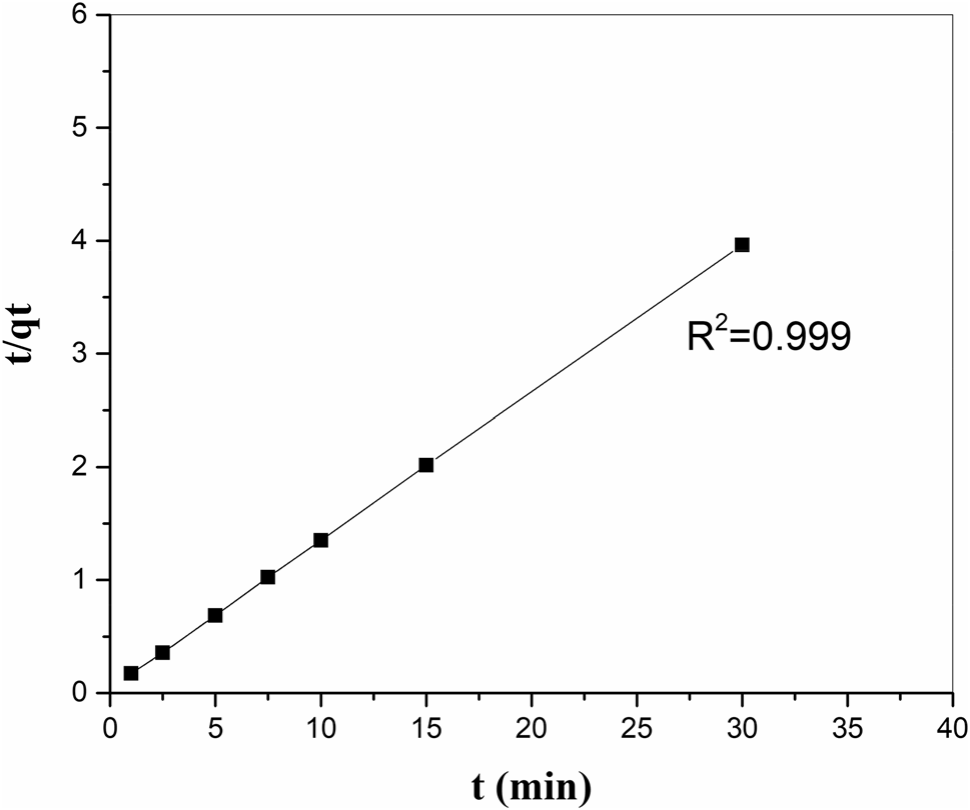
(PSO) plot for the removal of strontium ions onto zeolite/H_2_L.

To study the particular rate constants of the current adsorption reactions, we analyzed the kinetic data using pseudo-first-order, (PSO), Erovich, and power-function kinetic approaches. Kinetic factors were evaluated according to the linear graphs of the equations (Table 5). The data attained from the analysis of the current data indicated that strontium adsorption onto zeolite/H_2_L has been best described (*R*^*2*^ > 0.99) by a (PSO) kinetic equation (Fig. 9).

**Table 5:** The kinetic factors of strontium adsorption by the zeolite/H_2_L.

Kinetic models	Parameters	
Pseudo first-order $\mathrm{log}\left({q}_{t}-{q}_{e}\right)=\mathrm{log}{q}_{e}-\frac{{K}_{1}}{2.303}$ t	K_1_ (g/mg.min)	0.0401
	q_e_ (mg/g)	1.143
	R^2^	0.554
(PSO) $\frac{t}{{q}_{t}}=\frac{1}{{K}_{2}{q}_{e}^{2}}+\frac{1}{{q}_{e}}t$	K_2_ (g/mg.min)	0.453
	q_e_ (mg/g)	7.633
	R^2^	0.999
Elovich ${q}_{t}=a+2.303b\mathrm{log}t$	a (mg/g.min)	6.237
	b (g/mg)	0.476
	R^2^	0.759
Power function $\mathrm{log}{q}_{t}=\mathrm{log}a+b\mathrm{log}t$	a (mg/g)	6.214
	b (mg/g.min)	0.0714
	R^2^	0.736

The (PSO) equation is defined as follows^37^:

4 $$ \frac{t}{{q_{t} }}\, = \,\frac{1}{{K_{2} q_{e}^{2} }}\, + \,\frac{1}{{q_{e} }}t, $$

where q_e_ and q_t_ are the equilibrium adsorption capacity and the biosorption capacity at the time of t (mg/g), respectively, K_2_ is the rate constant of the (PSO) model (g/mg.min).

### Adsorption isotherms

**Figure 10:**
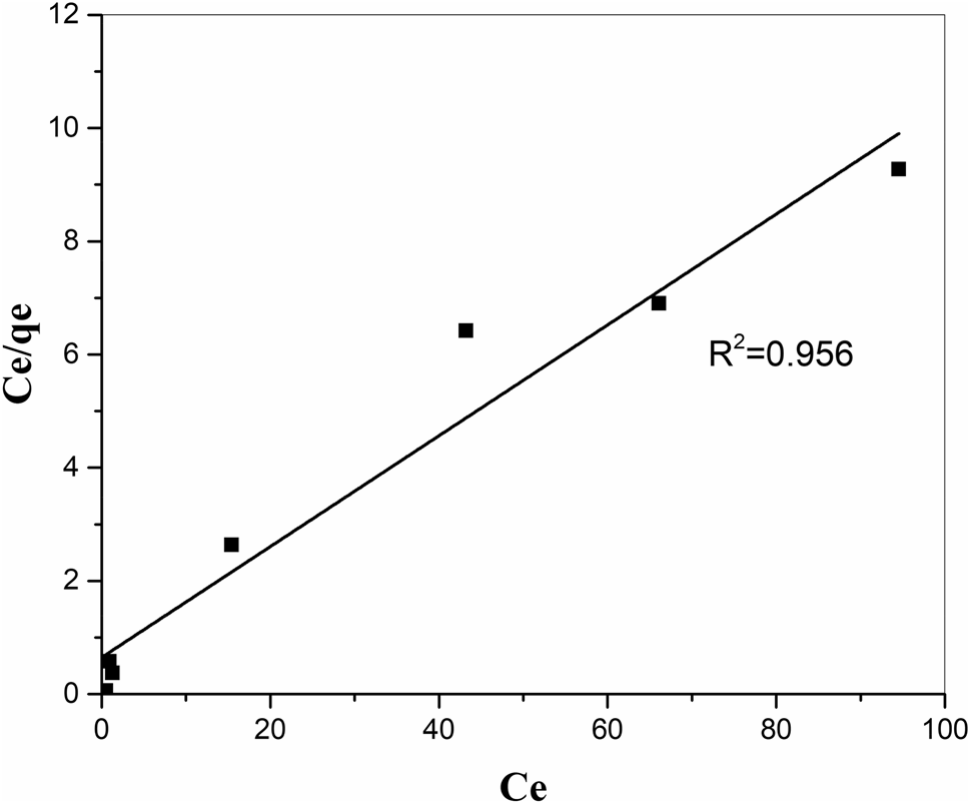
Langmuir isotherm scheme for the adsorption of strontium ions onto zeolite/H_2_L.

Isotherm equations are usually used to describe the adsorption equilibrium between the adsorbed ions and the dissolved ions in the liquid. In the present work, the obtained data were fitted with common isotherm models, including Langmuir, Freundlich, and Temkin. In Table 6, the values of the relevant isotherm parameters and their correlation factors (R^2^) are presented. High R^2^ is derived by fitting experimental results into the Langmuir isotherm model (Fig. 10, R^2^ = 0.956) compared to the Freundlich and Temkin isotherm models. Langmuir approach is often employed for monolayer sorption occurring on a homogeneous surface with identical sorption sites. Its linear type can be explained by the following correlation^34^:

5 $$ \frac{{{\text{C}}_{{\text{e}}} }}{{{\text{q}}_{{\text{e}}} }} = \frac{{1}}{{{\text{q}}_{{{\text{max}}}} {\text{b}}}} + \frac{{{\text{C}}_{{\text{e}}} }}{{{\text{q}}{}_{{{\text{max}}}}}}, $$

where q_max_ is the maximum adsorption capacity (mg/g), *q*_e_ is the amount of metal ion adsorbed per unit weight of sorbent (mg/g), *C*_e_ is the equilibrium concentration of the metal ion (mg/L), and b is the Langmuir constant (L/mg).

**Table 6:** The isothermal parameters of strontium adsorption by the zeolite/H_2_L.

Isotherms	Parameters	
Langmuir $\frac{{{\text{C}}_{{\text{e}}} }}{{{\text{q}}_{{\text{e}}} }} = \frac{{1}}{{{\text{q}}_{{{\text{max}}}} {\text{b}}}} + \frac{{{\text{C}}_{{\text{e}}} }}{{{\text{q}}{}_{{{\text{max}}}}}}$	q_max_	10.309
	b	0.150
	R^2^ R_L_	0.956 0.250
Freundlich ${\text{lnq}}_{{\text{e}}} = {\text{lnK}}_{{\text{f}}} + \frac{{1}}{{\text{n}}}{\text{lnC}}_{{\text{e}}}$	n	5.348
	K_f_	3.758
	R^2^	0.408
Temkin ${\text{q}}_{{\text{e}}} = \frac{{{\text{RT}}}}{{\text{b}}}{\text{ln(aC}}_{{\text{e}}} {)}$	a	114.269
	b	2.620
	R^2^	0.452

### Adsorption thermodynamics

**Figure 11:**
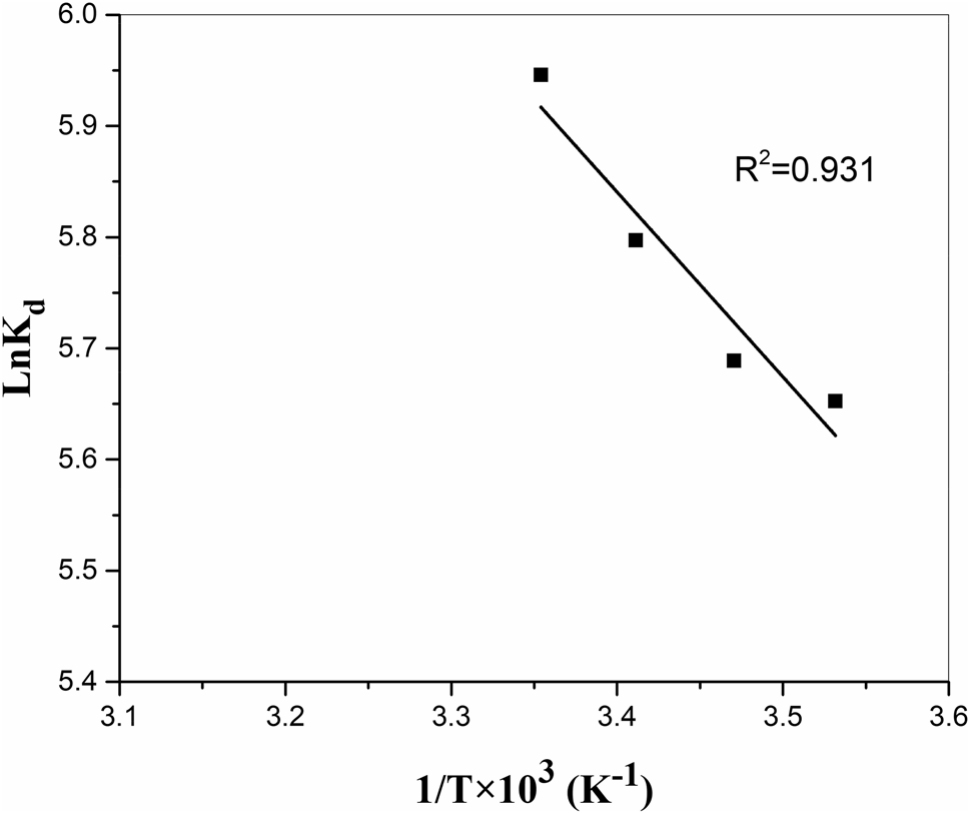
Changes of log K_d_ in terms of T^−1^ in the adsorption of strontium ion from aqueous solution.

To better understand the effect of temperature, the values of the thermodynamic factors, for instance, the Gibbs’ free energy (ΔGº), the standard enthalpy change (ΔHº), and the standard entropy change (ΔSº), were also studied. The enthalpy and entropy changes related to the metal ions adsorption process can be determined using the van't Hoff correlation presented here.

6 $$ \log K_{d} = - \frac{{\Delta H^{0} }}{2.303RT} + \frac{{\Delta S^{0} }}{2.303R}, $$

where K_d_ is the distribution coefficient, T is the absolute temperature (K), and R is the gas constant (0.0083 kJ/K.mol). Figure 11 shows the plot of ln*K*_d_ vs. 1/T for the adsorption of strontium ions, where a straight line is obtained. To determine enthalpy and entropy, the following Eqs. (7) and (8) are used:

7 $$ \Delta {\text{H}}^\circ = - R \times Slope $$

8 $$ \Delta {\text{S}}^\circ = R \times Intercept. $$

Based on ΔH° and ΔS°, the free energy variation (Δ*G*°) was also calculated (see Eq. (9)).

9 $$ \Delta G{^\circ } = \Delta H{^\circ } - T\Delta S{^\circ } . $$

The endothermic nature of the adsorption of the strontium ions onto the modified zeolite is suggested by the positive values of ΔH° (0.0138). The increasing randomness at the solid/solution interface during the adsorption process can be seen by the positive values of ΔS° (0.0955). Therefore, the adsorbent's affinity for this ion is indicated by the positive entropy of adsorption, and the values of ∆H° and ∆G° indicate that the adsorption of strontium on H_2_L/zeolite is an endothermic and spontaneous process (Table 7). The value of ΔG° (− 27.03 kJ/mol at 283 K and − 28.47 kJ/mol at 298 K, respectively) became more negative with the increase of temperature, which indicated that the adsorption process was more favorable at higher temperatures^36,38^. The values of − 400 < ΔG° < − 80 kJ/mol describes chemical adsorption while, the value of − 20 < ΔG° < 0 kJ/mol describes physical adsorption, and the value of − 80 < ΔG° < − 20 kJ/mol describes combination of physical and chemical adsorption^39–41^. Therefore, the values of calculated ΔG° confirmed the physicochemical adsorption property.

**Table 7:** Thermodynamics parameters of strontium adsorption by the zeolite/H_2_L.

ΔH^o^ (kJ/mol)	0.0138
ΔS^o^(kJ/mol.K)	0.0955
Temperature (K)	ΔG^o^(kJ/mol)
283	− 27.035
288	− 27.512
293	− 27.990
298	− 28.468

### Immobilization and leaching experiments

**Figure 12:**
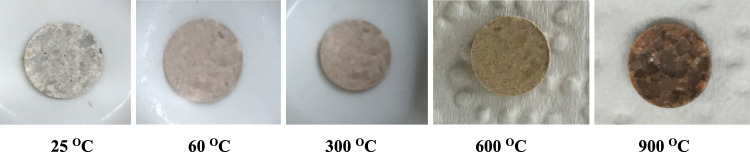
Pellets made from adsorbent after the adsorption of strontium ion after putting in the oven for 4 h at various temperatures.

First, 1 g of the modified adsorbent was dispersed in 50 mL of 600 mg/L Sr(NO_3_)_2_ solution and stirred for 24 h. Then the adsorbent was separated and dried. Using a hydraulic press, 5 tablets (0.2 g of the Sr-zeolite/H_2_L) were prepared and heated for 4 h at various temperatures of 25, 60, 300, 600, and 900 °C. With increasing temperature, the appearance of the pellets changed and darkened (Fig. 12). The change in the appearance of the samples at temperature of 25–300 °C is related to the release of water molecules that are into the zeolite structure, and the appearance change of the samples at temperatures higher than 300 °C is related to the burning, and structural decomposition of the Schiff base ligand.

Leaching tests were performed following the preparation of the pellets at various temperatures. To test the safety of immobilized samples, pellets were powdered and stirred for 24 h with 50 ml of a 1M NaCl solution. Then the amount of released strontium was evaluated. The results showed that the amount of strontium released from the adsorbed phase decreases with increasing heat treatment temperature (Table 8).

**Table 8:** Leaching test results of thermal treatment for samples.

Temperature (°C)	The strontium concentration trapped in the adsorbent (mg/L)	The strontium concentration released in the solution phase (mg/L)	The percentage of strontium released in solution phase (%)	The percentage of stabilized strontium (%)
25	248	130.4	52.6	47.4
60	248	110.8	44.7	55.3
300	248	50.7	20.4	79.6
600	248	14.3	5.8	94.2
900	248	4.0	1.6	98.4

## Conclusions

Studies conducted on natural zeolite of the Sabzevar region show that this type of zeolite has a crystalline structure and is the type of sodium clinoptilolite, and its particle size is equal to 29.49 nm. Examining the ion exchange properties of the zeolite showed that it can be used as an ion exchange of strontium from aqueous solutions. The Adsorption process was dependent on the environmental pH and in the range of 4–6 showed the best performance. To improve the performance of zeolite adsorption from Schiff base ligand synthesized to encapsulate in the zeolite pores was used. To compare the efficiency of adsorption of strontium ions onto unmodified and modified zeolite showed that the ligand present in the zeolite structure significantly increases the adsorption efficiency of strontium ions. So that the strontium ion in optimum conditions is almost completely removed from the medium. Kinetic, isotherm, and thermometric studies revealed that the removal process follows the (PSO) model, Langmuir adsorption isotherm, and endothermic process, respectively. Given that the adsorption of strontium ion on modified zeolite was a significant amount, to verify that the adsorbent can also be used as an appropriate medium to stabilize the strontium ion, the experiments to stabilize the strontium ion were performed at different temperatures. The results showed that Sr-zeolite/H_2_L retains strontium after heat treatment at 900 °C and can be used as a suitable matrix for stabilizing and maintaining strontium ions. Hence the differences in zeolite structure and composition determine the final safety of the strontium retention. Using the results of this study and similar works, these resources can be used in wastewater purification, especially wastes resulting from nuclear activities. The main advantages of the use of zeolite is easy to use, inexpensive, and optimal compatibility with the environment and the lack of contaminating the environment.

## Data Availability

The datasets used and/or analyzed during the current study are available from the corresponding author on reasonable request.
